# Evaluation of DawaPlus 3.0 and DawaPlus 4.0, deltamethrin–PBO combination nets against pyrethroid-resistant *Anopheles culicifacies* in experimental huts in India

**DOI:** 10.1186/s12936-020-3119-x

**Published:** 2020-01-23

**Authors:** Kasinathan Gunasekaran, Sudhansu Sekhar Sahu, Tharmalingam Vijayakumar, Swaminathan Subramanian, Manju Rahi, Purushothaman Jambulingam

**Affiliations:** 1 Vector Control Research Centre, Indian Council of Medical Research, Indira Nagar, Pondicherry, 605006 India; 2 Indian Council of Medical Research, New Delhi, India

**Keywords:** *Anopheles culicifacies*, DawaPlus 3.0, DawaPlus 4.0, Experimental huts, India, Piperonyl butoxide

## Abstract

**Background:**

The development of resistance in vectors is one of the major impediments for malaria control. Adding synergists to insecticides has proven to be an alternative choice for controlling resistant mosquitoes. DawaPlus 3.0 and DawaPlus 4.0 are new long-lasting insecticidal nets (LLINs) in which deltamethrin and a synergist, piperonyl butoxide (PBO) are added into filaments and their efficacy was tested against resistant malaria vector, *Anopheles culicifacies* in experimental huts in India.

**Methods:**

The performance of two trial nets in terms of deterrence induced exiting, blood-feeding inhibition and mortality of *An. culicifacies* was compared with DawaPlus 2.0 and untreated net.

**Results:**

There was a significant reduction in entry, blood feeding and mortality (p < 0.05) and increase in exit rates of *An. culicifacies* in the treatment arms compared to untreated arm. But, both candidate LNs washed 20 times could not perform better than the washed reference net (DawaPlus 2.0). Cone bioassay results showed that all the treatment arms (both washed and unwashed) produced < 80% mortality of *An. culicifacies* before and after hut evaluation.

**Conclusions:**

DawaPlus 3.0 and DawaPlus 4.0 with their current specification may not be as effective as required to control the resistant vector, *An. culicifacies,* in east-central India.

## Background

Vector control measures using pyrethroids are the recent and most effective method for combating the malaria vectors in many parts of the world [1]. In India, the main vector control tools adopted are long-lasting insecticidal nets (LLINs) and indoor residual spraying (IRS) [2]. The development of resistance in vectors to insecticides is one of the major impediments for malaria control. Recent studies have confirmed the development of resistance by major malaria vectors to the commonly used pyrethroids [3, 4]. Currently, efforts have focussed on initiating newer control strategies using combination of different chemical compounds that could be effective against pyrethroid resistance. Adding synergists to insecticides has proven to be an alternative choice for controlling resistant mosquitoes [5, 6]. Piperonyl butoxide (PBO), a synergist, when used in combination with insecticides (carbamates, pyrethrins, and pyrethroids) can enhance their potency and protect the co-applied insecticide from metabolic attack thus allowing them to reach their biochemical targets [7, 8].

Combination or mixture LLINs with PBO could be effective against resistant mosquitoes whose resistance is based on oxidative metabolism [5]. DawaPlus 3.0 and DawaPlus 4.0 are among the new generation mosquito nets that were recommended by WHOPES for Phase II evaluation. These LLINs are treated with deltamethrin and the oxidase synergist PBO [9, 10]. The candidate LLIN, DawaPlus 3.0 from Tana Netting, UAE, is a combination LLIN comprised of side panels made of knitted poly-filament polyester fibres (42 g/m^2^) coated with 2.5 g ai/kg deltamethrin (105 mg/m^2^), and roof made of polyethylene (40 g/m^2^) incorporating 3 g ai/kg deltamethrin (120 mg ai/m^2^) and 12 g/kg PBO (480 mg/m^2^). The second candidate LLIN, DawaPlus 4.0 also from Tana Netting, is a mixture net comprised of side and roof panels made of polyethylene (40 g/m^2^) incorporating 3 g ai/kg deltamethrin (120 mg ai/m^2^) and 12 g/kg PBO (480 mg/m^2^) [9].

The current study evaluated the efficacy of DawaPlus 3.0 and DawaPlus 4.0 in experimental huts, against deltamethrin resistant natural population of *Anopheles culicifacies* in terms of deterrence, induced exiting, blood-feeding inhibition and mortality in India during April 2016 to December 2016.

## Methods

### Study area

The experimental hut evaluation of DawaPlus 3.0 and DawaPlus 4.0 was conducted in seven experimental huts constructed in Kandhaguda village of Pandripani Community Health Centre (CHC) in Malkangiri district of Odisha State, a part of east-central India. The specifications of the experimental huts were according to the WHO guidelines [11].

The CHC has been highly endemic for malaria since many years. *Plasmodium falciparum* is the major parasite species, contributing to > 90% of the total malaria cases [2]. Transmission of malaria occurs throughout the year, but there are seasonal fluctuations; the incidence is low in summer, increases in rainy and peaks during cooler months*. Anopheles fluviatilis* and *Anopheles culicifacies* are the major malaria vectors in the area [2]. *An. fluviatilis* is abundant during September–February (peaks during November–December) and *An. culicifacies* during March–September (peaks during July–August) [2]. While, *An. fluviatilis* was susceptible to DDT and pyrethroids; *An. culicifacies* was resistant to both [3].

### Acclimatization and hut suitability

Prior to hut evaluation, an adult volunteer slept under an untreated mosquito net in each of the 7 huts from dusk to dawn for a period of 1 month to make the huts suitable for evaluation in terms of attracting mosquitoes comparable to village huts. Approval was obtained from the Indian Council of Medical Research (ICMR)-Vector Control Research Centre (VCRC) Human Ethics Committee, Puducherry for engaging adult human volunteers.

Suitability of huts was assessed by comparing the indoor resting density of *An. culicifacies* in the experiment huts with that in the village-huts and by assessing recovery and scavenging rates for 1 month prior to hut evaluation, which has been described elsewhere [12].

### Experimental arms

Seven comparison arms viz., Arm 1: Untreated polyester net (negative control), Arm 2: Unwashed DawaPlus 2.0 (positive control), Arm 3: DawaPlus 2.0 washed 20 times (positive control), Arm 4: Unwashed DawaPlus 3.0, Arm 5: DawaPlus 3.0 washed 20 times, Arm 6: Unwashed DawaPlus 4.0 and Arm 7: DawaPlus 4.0 washed 20 times were tested in experimental huts for their efficacy on *An. culicifacies* in terms of deterring entry, inhibiting blood-feeding, inducing mortality and repelling or driving mosquitoes out of huts. Primary comparison was made with both positive and negative controls. Before start of the evaluation, the nets were coded. LLINs of Arm 2, 4 and 6 were washed 20 times (8 nets in each Arm) as per the WHO washing protocol [11].

Bioassays and chemical analysis were done on the same nets on adjacent pieces. Cone bioassays were carried out on nets before any wash, after 10 washes and washing after 20 times using laboratory reared, susceptible fully-fed *Anopheles stephensi.* Prior to and after the hut evaluation, cone-bioassays were carried out exposing wild caught pyrethroid-resistant *An. culicifacies* on one net randomly selected from the six replicate nets per arm. The details of bioassay, chemical analysis, process of selection of volunteers, preparation of nets and rotation of treatments and volunteers have been described elsewhere [12].

### Collection and processing of mosquitoes

In the evening, white cloths were spread inside the room and verandah. The gutters were filled with water to prevent entering of ants and scavenging. The volunteers slept under the net from 19.00 to 05.30 h in the assigned huts. The next day morning, live and dead mosquitoes were collected separately from inside the net, veranda and the hut, labelled and maintained separately by hut and collection sites (veranda trap, room, inside net) for further processing.

### Statistical analysis

The mean per-hut density (PHD) of *An. culicifacies* before hut evaluation was compared between experimental huts and village huts using one way ANOVA. The number of occasions that recorded hut entry of the vector species was compared between different arms using χ^2^ test. For statistical analysis, the number of mosquitoes entered the huts, the proportion of mosquitoes that exited early, the proportion that were killed within the hut (immediate mortality) or after 24 h (delayed mortality) and the proportion that successfully blood fed were compared by species with the hut as the repeat unit. The statistical procedure was logistic regression for proportional data and negative binomial regression for numeric data; adjustments were made for the effect of hut and sleeper. For overall comparison, untreated net (negative control) was kept as reference category. Comparison between the candidate LNs and the positive control was made from the 95% confidence intervals (CI) of the incidence rate ratio (IRR) or odds ratios, as applicable. Comparisons were also made by excluding negative control from the grouped data and keeping the positive control (unwashed DawaPlus 2.0) as reference category.

## Results

### Acclimatization and hut suitability

The mean PHD of *An. culicifacies* was 10.3 ± 2.58 in experimental huts and 12.2 ± 2.6 in village huts; the two mean values did not differ significantly (p > 0.05). The recovery rates of *An. culicifacies* varied from 80% (359/450) to 86% (515/600). The scavenging was almost nil on all occasions except on a few in the beginning when the scavenging rate varied from 1.2 to 11.4%.

### Species composition

In total, 42 collections were carried out for each of the seven arms and totally 816 mosquitoes were collected. *Anopheles culicifacies* was the predominant species (45.8%). *Anopheles fluviatilis* formed only 0.24%, other anophelines (*Anopheles subpictus* and *Anopheles vagus*) 31.0% and culicines 22.9%.

### Entry

Over 42 collections, conducted during 7 weeks period, 6 collections per week, the mean hut entry ± SE of *An. culicifacies* was 6.28 ± 0.52, 0.4 ± 0.1, 0.73 ± 0.12, 0.24 ± 0.07, 0.45 ± 0.09, 0.29 ± 0.08 and 0.5 ± 0.11, respectively for the experimental arms: untreated polyester net (negative control), unwashed DawaPlus 2.0 (positive control), DawaPlus 2.0 washed 20 times (positive control), unwashed DawaPlus 3.0, DawaPlus 3.0 washed 20 times, unwashed DawaPlus 4.0 and DawaPlus 4.0 washed 20 times (Table 1).

**Table 1:** Hut entry of *An. culicifacies* in treated and untreated arms with the results of negative binomial regression analysis

Experiment arms	Number of collections	Number entered (entry)^a^	Range	Mean entry ± SE	Incidence rate ratio (IRR)	95% CI	p
Untreated polyester net (negative control)	42	264	0–16	6.28 ± 0.07	1.00^b^		
Unwashed DawaPlus 2.0 (positive control)	42	17	0–1	0.40 ± 0.09	0.064	0.038–0.107	0.000
DawaPlus 2.0 washed 20 times (positive control)	42	31	0–3	0.73 ± 0.08	0.117	0.078–0.175	0.000
Unwashed DawaPlus 3.0	42	10	0–1	0.24 ± 0.11	0.037	0.019–0.072	0.000
DawaPlus 3.0 washed 20 times	42	19	0–2	0.45 ± 0.10	0.071	0.044–0.117	0.000
Unwashed DawaPlus 4.0	42	12	0–1	0.29 ± 0.12	0.045	0.025–0.082	0.000
DawaPlus 4.0 washed 20 times	42	21	0–2	0.50 ± 0.52	0.079	0.049–0.127	0.000

^a^Out of the total 42 collections in each arm; *SE* standard error

^b^Untreated polyester net (negative control) was used as reference category for the analysis

Overall, hut entry of *An. culicifacies* differed significantly (χ^2^ = 227.63, df = 6, p < 0.0001) between the experimental arms. Compared to the negative control, hut entry was significantly lower in all treated arms (p < 0.05). Using the 95% CIs of the Incidence rate ratios (IRRs), hut entry was compared between the candidate LNs (DawaPlus 3.0 and DawaPlus 4.0) and the positive control (DawaPlus 2.0). The entry was significantly lower with unwashed DawaPlus 3.0 than that with washed DawaPlus 2.0, but not with unwashed DawaPlus 2.0. Washed DawaPlus 3.0 did not differ significantly from DawaPlus 2.0 washed or unwashed (Table 1). The deterrent effect (preventing hut entry) of DawaPlus 4.0 washed or unwashed was comparable with that of DawaPlus 2.0 washed or unwashed.

When Poisson regression analysis was done after eliminating the negative control and by keeping the positive control (unwashed DawaPlus 2.0) as reference category, overall, there was a significant difference in hut entry of *An. culicifacies* among the treatment arms (χ^2^ = 14.77, df = 5, p = 0.011). This significant difference could be the reflection of a higher entry with washed DawaPlus 2.0 than the unwashed (positive control). However, the difference was at its statistical limit (p = 0.047). Except this difference, there was no significant difference between the candidate LN, DawaPlus 3.0 or DawaPlus 4.0, washed or unwashed and the positive control.

### Exit (induced exophily)

The number of *An. culicifacies* exited on each day of collection in each arm was pooled together for 7 weeks. Overall, the exit rate in the treated arms ranged from 52.9% (Unwashed DawaPlus 2.0) to 70% (Unwashed DawaPlus 3.0). The exit rate in the untreated arm was 23.9% (Table 2). There was a significant difference in the exit rate among the seven experiment arms (χ^2^ = 42.58, p = 0.000) with a greater exit in all the treated arms than the untreated arm. The 95% CI for the odds ratio indicated a comparable exit rate between DawaPlus 3.0 washed or unwashed and DawaPlus 2.0, washed or unwashed. Similarly, the exit rate with DawaPlus 4.0 washed or unwashed was not significantly different from DawaPlus 2.0 washed or unwashed.

**Table 2:** Exit rate of *An. culicifacies* recorded in treated and untreated arms with the results of negative binomial regression analysis

Experiment arms	No. of collections	Number entered^a^	Number exited	Exit rate (%)	Odds ratio	95% CI	p
Untreated polyester net (negative control)	42	264	63	23.9	1.00^b^		
Unwashed DawaPlus 2.0 (positive control)	42	17	9	52.9	3.589	1.329–9.694	0.012
DawaPlus 2.0 washed 20 times (positive control)	42	31	18	58.1	4.418	2.051–9.517	0.000
Unwashed DawaPlus 3.0	42	10	7	70.0	7.444	1.869–29.644	0.004
DawaPlus 3.0 washed 20 times	42	19	12	63.2	5.469	2.065–14.488	0.001
Unwashed DawaPlus 4.0	42	12	7	58.3	4.467	1.369–14.565	0.013
DawaPlus 4.0 washed 20 times	42	21	12	57.1	4.254	1.713–10.562	0.002

^a^Out of the total 42 collections in each arm

^b^Untreated polyester net (negative control) was used as reference category for the analysis

There was also some amount of exit in the untreated arm, might be due to the innate behaviour of the vector species. However, the significantly greater exit of *An. culicifacies* observed with the candidate LNs (DawaPlus 3.0 & DawaPlus 4.0) and the positive control (DawaPlus 2.0) could be the treatment-induced exophily.

### Blood feeding rate

The percentage of blood-fed *An. culicifacies* in the seven experiment arms is given in Table 3. The feeding rate in the untreated arm (negative control) was 93.9%. Among the treated arms, while the feeding rate was zero with unwashed DawaPlus 3.0, it was relatively lower with washed DawaPlus 3.0 (42.1%) and washed and unwashed DawaPlus 4.0 (66.7%) than the positive controls, (DawaPlus 2.0: unwashed, 76.5% and washed, 67.7%). Thus, there was a complete inhibition of blood-feeding with unwashed DawaPlus 3.0 and marginally a higher inhibition with washed DawaPlus 3.0 compared to the positive control. Both washed and unwashed DawaPlus 4.0 also showed slightly a higher inhibition than DawaPlus 2.0.

**Table 3:** Feeding rate of *An. culicifacies* in treated and untreated arms with the results of negative binomial regression analysis

Experiment arms	No. of collections	Number entered^a^	Number fed	% fed	Odds ratio	95% CI	p
Untreated polyester net (negative control)	42	264	248	93.9	1.00^b^		
Unwashed DawaPlus 2.0 (positive control)	42	17	13	76.5	0.209	0.061–0.717	0.013
DawaPlus 2.0 washed 20 times (positive control)	42	31	21	67.7	0.135	0.055–0.335	0.000
Unwashed DawaPlus 3.0**	42	10	0	0	–	–	–
DawaPlus 3.0 washed 20 times	42	19	8	42.1	0.047	0.016–0.133	0.000
Unwashed DawaPlus 4.0	42	12	8	66.7	0.129	0.035–0.474	0.002
DawaPlus 4.0 washed 20 times	42	21	14	66.7	0.129	0.045–0.364	0.000

**No outcome of logistic regression analysis as the number fed was zero in this arm

^a^Out of the total 42 collections in each arm

^b^Untreated polyester net (negative control) was used as reference category for the analysis

The blood feeding rate differed significantly among the experiment arms (logistic regression: χ^2^ = 52.44, df = 5, p = 0.000). Compared to the untreated arm, the feeding rate was significantly (p < 0.05) lower in all the six treated arms (outcome of the analysis could not be shown for unwashed DawaPlus 3.0 as feeding rate with this arm was zero). The 95% CI for the odds ratios showed that the feeding rate with DawaPlus 3.0 washed or unwashed and DawaPlus 4.0 (washed or unwashed) was not significantly different than DawaPlus 2.0 (washed or unwashed) (Table 3).

### Mortality

The immediate, delayed and total mortality rates of *An. culicifacies* are given in Table 4. The immediate mortality was zero in all the arms and hence the delayed mortality was also the total mortality. With the untreated arm, the delayed/total mortality was zero. Among the treated arms, DawaPlus 3.0 and DawaPlus 4.0 (washed or unwashed) caused relatively higher mortality than the positive control, DawaPlus 2.0 (washed or unwashed).

**Table 4:** Mortality rate of *An. culicifacies* in treated and untreated arms with the results of negative binomial regression analysis

Experiment arms	Number entered^a^	Immediate mortality (%)	Delayed mortality (%)	Total mortality (%)	Odds ratio	95% CI	p
Untreated polyester net (negative control)	264	0.0	0.0	0.0	–	–	–
Unwashed DawaPlus 2.0 (positive control)	17	0.0	17.6	17.6	1.00^b^	–	–
DawaPlus 2.0 washed 20 times (positive control)	31	0.0	25.8	25.8	1.623	0.368–7.159	0.522
Unwashed DawaPlus 3.0	10	0.0	50.0	50.0	4.667	0.804–27.077	0.086
DawaPlus 3.0 washed 20 times	19	0.0	31.6	31.6	2.154	0.444–10.438	0.341
Unwashed DawaPlus 4.0	12	0.0	41.7	41.7	3.333	0.612–18.149	0.164
DawaPlus 4.0 washed 20 times	21	0.0	33.3	33.3	2.333	0.499–10.907	0.282

^a^Out of the total 42 collections in each arm

^b^Since, no mortality was recorded with untreated net (negative control), it was removed from the grouped data. Instead, the positive control, unwashed DawaPlus 2.0 was used as reference category for logistic regression analysis

Since, the untreated arm produced no mortality, it was removed from the grouped data and the positive control was kept as reference category for logistic regression analysis. Overall, there was no significant difference in delayed/total mortality among the six experiment arms (excluding the negative control) (χ^2^ = 4.20, df = 5, p = 0.521). Further, DawaPlus 3.0 washed or unwashed and the reference category, DawaPlus 2.0 washed or unwashed caused comparable mortality. Similarly, the mortality caused by DawaPlus 4.0 washed or unwashed was not significantly different from DawaPlus 2.0, washed or unwashed (from the 95% CI for the odds ratios).

The candidate LNs, DawaPlus 3.0 or DawaPlus 4.0, washed or unwashed were not significantly different from the positive control, DawaPlus 2.0 in terms of induced exophily (χ^2^ = 0.95, df = 5, p = 0.966), blood feeding rate (χ^2^ = 5.27, df = 4, p = 0.260) and delayed/total mortality (χ^2^ = 4.20, df = 5, p = 0.521) indicating a comparable performance of the candidate LNs with the positive control (Additional file 1: Table S1).

### Cone-bioassay mortality and insecticide content

Prior to washing and after 10 and 20 washes, the mortality of *An. stephensi* (susceptible to deltamethrin) in cone-bioassays was 100% with all the three LNs, DawaPlus 3.0, DawaPlus 4.0 and DawaPlus 2.0 and the mortality was zero with the untreated net (Table 5).

**Table 5:** Results of cone-bioassays

Experiment arms	Before any wash		After 10 washes		After 20 washes		Prior to hut evaluation		After hut evaluation	
	NE	CM (%)	NE	CM (%)	NE	CM (%)	NE	CM (%)	NE	CM (%)
Untreated polyester net (negative control)	50	0	50	0	50	0	50	0	50	0
Unwashed DawaPlus 2.0 (positive control)	50	100	50	100	50	100	50	32	50	40
DawaPlus 2.0 washed 20 times (positive control)	50	100	50	100	50	100	50	26	50	34
Unwashed DawaPlus 3.0	50	100	50	100	50	100	50	48	50	50
DawaPlus 3.0 washed 20 times	50	100	50	100	50	100	50	34	50	70
Unwashed DawaPlus 4.0	50	100	50	100	50	100	50	48	50	54
DawaPlus 4.0 washed 20 times	50	100	50	100	50	100	50	4	50	36

Prior to and after experiment hut evaluation, *An. culicifacies* was used for bioassay whereas before any wash and after washes *An. stephensi* was used for the assay

*NE* number exposed, *CM* corrected mortality

Before the hut trial, the cone-bioassay mortality of *An. culicifacies* was 48% on unwashed DawaPlus 3.0 and 34% on DawaPlus 3.0 washed 20 times. After the hut trial, the corresponding mortality was 50.0% and 70.0%. When analysed according to panels, prior to hut trial, the mortality was 80.0% on roof and 40.0% on sides of unwashed DawaPlus 3.0 and after hut trial, the mortalities were 40.0% and 52.5%, respectively. In the case of DawaPlus 3.0 washed 20 times, prior to hut trial, the mortality was 40% on roof and 32.5% on sides and after hut trial, the mortality was 70% on both roof and sides (Table 5). The difference in mortality prior to and after the hut trial could be due to the heterogeneity of the field population of *An. culicifacies* in terms of their response to the insecticide.

The results of chemical analysis are given in Tables 6 and 7. The mean deltamethrin content of two net samples of side panels of unwashed Dawaplus 3.0 (2.50 and 2.58 g/kg) complied with the target dose of 2.5 g/kg ± 25% [1.9–3.1 g/kg]. After 20 washes, the deltamethrin content was 0.96 g/kg with a retention of 37%. After the hut trial, the deltamethrin content did not diminish, as it was 2.47 g/kg in the side panels of unwashed DawaPlus 3.0 and 0.94 g/kg in the side panels of DawaPlus 3.0 washed 20 times. In the 2 net samples of roof panel of unwashed DawaPlus 3.0, the mean deltamethrin content was 2.38 and 2.33 g/kg and complied with the target dose of 3.0 g/kg ± 25% [2.3–3.8 g/kg]. After 20 washes, the deltamethrin content was same as the baseline with 100% retention (Table 6). After the hut trial also, there was no loss of deltamethrin content.

**Table 6:** Deltamethrin content in net samples before and after washing, and after hut trial

Treatment	AI content (g/kg) before washing	AI content (g/kg) after 10 washes	AI content (g/kg) after 20 washes	AI retention (% of wash 0)	AI content (g/kg) after hut trial	Compliance of baseline samples with WHO specification	AI within-net variation (RSD)
Unwashed DawaPlus 3.0 roof	2.38	–	–	–	2.33	Yes	–
DawaPlus 3.0 roof washed 20 times	2.33	2.33	2.33	100%	2.38	Yes	–
Unwashed DawaPlus 3.0 sides	2.50	–	–	–	2.47	Yes	7.1%
DawaPlus 3.0 sides washed 20 times	2.58	1.81	0.96	37%	0.94	Yes	2.9%
Unwashed DawaPlus 4.0	2.32	–	–	–	2.34	Yes	3.8%
DawaPlus 4.0 washed 20 times	2.36	2.26	2.16	91%	2.24	Yes	5.8%
Unwashed DawaPlus 2.0	2.08	–	–	–	2.02	Yes	2.4%
DawaPlus 2.0 washed 20 times	2.07	1.18	0.74	36%	0.64	Yes	3.9%
Untreated polyester net	<0.01	–	–	–	<0.01

**Table 7:** Piperonyl butoxide content in LNs before and after washing, and after hut trial

Treatment	AI content (g/kg) before washing	AI content (g/kg) after 10 washes	AI content (g/kg) after 20 washes	AI retention (%)	AI content (g/kg) after hut trial	Compliance with target dose	AI within-net variation (RSD)
Unwashed DawaPlus 3.0 roof	7.9	–	–	–	6.4	No	–
DawaPlus 3.0 roof washed 20 times	8.1	6.2	5.2	64%	4.7	No	–
Unwashed DawaPlus 4.0	9.7	–	–	–	9.3	Yes	13%
DawaPlus 4.0 washed 20 times	9.9	7.3	6.0	61%	5.9	Yes	12%
Untreated polyester net	<0.1	–	–	–	<0.1		

The cone-bioassay mortality of *An. culicifacies*, prior to hut trial, was 48% on unwashed DawaPlus 4.0 and 4% on DawaPlus 4.0 washed 20 times. After hut trial, the mortality was 54.0 and 36.0%, respectively (Table 5). The mean deltamethrin content in 2 unwashed DawaPlus 4.0 net samples was 2.32 and 2.36 g/kg which complied with the target dose of 3.0 g/kg ± 25% [2.3–3.8 g/kg]. The deltamethrin content after 20 washes was 2.16 g/kg corresponding to a retention of 91%. After hut trial, there was no decline in deltamethrin content, as it was 2.34 g/kg for the unwashed and 2.24 g/kg for the 20 times washed DawaPlus 4.0. Despite the availability of the required quantity of deltamethrin content even after 20 washes and after the hut trial, cone-bioassay mortality of *An. culicifacies* was lesser than 60% (Table 6).

Prior to hut trial, the cone-bioassay mortality was 32% on unwashed DawaPlus 2.0 and 26% on DawaPlus 2.0 washed 20 times and after the hut trial the corresponding mortality was 40% and 34% (Table 5). The mean deltamethrin content in the two unwashed DawaPlus 2.0 was 2.08 and 2.07 g/kg that complied with the target dose of 2.0 g/kg ± 25% [1.5–2.5 g/kg]. After 20 washes, the deltamethrin content was 0.74 g/kg with a retention of 36%. After the hut trial, the deltamethrin content did not decrease, as it was 2.02 g/kg for the unwashed and 0.64 g/kg for the 20 times washed DawaPlus 2.0. Like DawaPlus 3.0 and DawaPlus 4.0, the cone-bioassay mortality on DawaPlus 2.0 washed or unwashed was very low (≤ 40%) prior to and after hut trial (Table 5).

The piperonyl butoxide (PBO) content in the two net samples of roof panel of unwashed DawaPlus 3.0 was 7.9 and 8.1 g/kg that did not comply with the target dose of 11.0 g/kg ± 25% [8.3–13.8 g/kg]; slightly under-dosed. The mean PBO content in the two unwashed DawaPlus 4.0 was 9.7 and 9.9 g/kg, complying with the target dose of 11.0 g/kg ± 25% [8.3–13.8 g/kg]. The PBO content was 6.0 g/kg after 20 washes with a retention of 61%. After the hut trial, there was no reduction in PBO content, as it was 9.3 g/kg for the unwashed and 5.9 g/kg for the 20 times washed DawaPlus 4.0 (Table 7). The deltamethrin and PBO content in the untreated polyester net before washing and after the hut trial was lower than the limit of quantification (< 0.01 g/kg for deltamethrin and < 0.1 g/kg for PBO).

### Side effects

Interview of the 14 volunteers revealed that no volunteer suffered either from nose irritation or itching of face and hands etc. All volunteers who slept under nets of different arms stated that they did not get any odour from the nets. The benefit perceived by them was the reduced mosquito bites in the huts and undisturbed night sleep throughout the study period.

## Discussion

Development of insecticide resistance is one of the major challenges to manage the vectors of malaria all over the world. Currently, pyrethroids are the only class of insecticide that can make vector control more feasible. The existing effective tools that are using pyrethroids are LLINs and IRS [13] which have drastically reduced malaria burden in many endemic countries during the last decade [14, 15]. Therefore, it is indispensable to safeguard pyrethroids as long as feasible because, so far, no other insecticide class has replaced pyrethroids for its effectiveness, safety, cost effectiveness, acceptability for LLIN and IRS [16, 17]. However, with the massive use of pyrethroids in both public health and agriculture sectors, there were reports of rapid development of pyrethroid resistance among malaria vectors which is a serious concern undermining the effectiveness of both the tools (LLIN and IRS) [16–19]. In this scenario, combining a synergist PBO with pyrethroids on net fibres could be a promising way to fight the resistant vectors.

Currently, there are two types of new generation LNs (permethrin with PBO and deltamethrin with PBO) targeting resistance vector mosquitoes [6]. DawaPlus 3.0 is one such long-lasting combination net with deltamethrin on side panels and a mixture of deltamethrin and PBO on roof panel. The DawaPlus 4.0 is also a knitted fabric LN, in which technical deltamethrin and technical PBO are incorporated on all side panels and roof. The current study evaluated the performance of DawaPlus 3.0 and DawaPlus 4.0 in comparison to DawaPlus 2.0 against the malaria vector *An. culicifacies,* which has developed resistance to multiple insecticides including pyrethoids throughout India [3, 4]. The study showed that overall, the performance of unwashed or washed (20 times) DawaPlus 3.0 and DawaPlus 4.0 was significantly higher than the negative control and comparable to the reference net (DawaPlus 2.0) in terms of deterrence, induced exophily, blood feeding inhibition and induced mortality. The cone bioassays conducted before hut evaluation showed that PBO did not show a significant synergistic action on roof panel of washed DawaPlus 3.0, as mortality of *An. culicifacies* was only 40% compared to 32.5% mortality on side panels that did not have PBO. Overall, DawaPlus 3.0 and DawaPlus 4.0 unwashed and washed 20 times did not cause ≥ 80% mortality of the pyrethroid-resistant *An. culicifacies* in cone bio-assays before and after hut evaluation, resulting in non-compliance to the World Health Organization (WHO) criteria for bio-efficacy [11]. In spite of adding PBO with deltamethrin, the performance of both DawaPlus 3.0 and DawaPlus 4.0 was not better than the reference net indicating that adequate protection against biting of resistant mosquitoes may not be possible with these PBO nets.

In many other experimental hut trials, combined PBO pyrethroid LNs inhibited blood feeding and raised mortality against pyrethroid-resistant malaria vectors [6, 20, 21]. Permanet 3.0, a combination LN incorporated with deltamethrin and PBO on top panel has been widely evaluated against pyrethroid-resistant *Anopheles gambiae* sensu lato populations in different ecological settings and the results showed an equal or better performances compared to PermaNet 2.0 [6]. However, in the current study, synergistic effect of PBO added with deltamethrin was not observed as the performance of PBO net was similar to non-PBO reference net. Further, inclusion of PBO on all panels of DawaPlus 4.0 did not enhance its performance over either DawaPlus 3.0 or the reference net. Similar to these results, the deltamethrin–PBO combination net was not found effective against pyrethroid-resistant *An. gambiae* and *Culex quinquefasciatus* in Benin [22]. Another study conducted in Southern Benin showed that the combined permethrin–PBO net was not a solution to control the pyrethroid-resistant *Anopheles coluzzii* [23]. Therefore, pyrethroid–PBO mixture nets could not always be a promising strategy against pyrethroid-resistant malaria vector populations. However, the susceptibility bioassays conducted recently in the current study area showed that when the resistant population of *An. culicifacies* was exposed to PBO prior to the exposure to deltamethrin, 100.0% susceptibility got restored [24]. But, when LLINs are treated with PBO and pyrethroids (mixture or combination nets), their effectiveness against the resistant vector species was not up to the desired level, as evidenced from the results of the current study. This was further supported from the results of the cone bioassays conducted with pyrethriod susceptible *An. stephensi* and resistant *An. culicifacies* before the hut evaluation. The mortality of *An. stephensi* when exposed to DawaPlus 2.0 (positive control), DawaPlus 3.0 and DawaPlus 4.0 unwashed and washed 20 times was 100%, whereas for *An. culicifacies* the mortality was < 50%. The underperformance of DawaPlus 3.0 and DawaPlus 4.0 could be related to some technical issues such as formulation, treatment technology of nets to ensure adequate dosage of PBO, bio-availability of PBO to mosquitoes, PBO-non target mechanism of resistance etc. Therefore, further improvement of the formulations (mixtures or combinations) and treatment technology are needed to ensure bioavailability of insecticides and the synergist (PBO) at adequate dosages and to avoid excessive losses during washing.

## Conclusions

Contrary to our findings, the phase II trials conducted in the two countries viz., Tanzania and Burkina Faso, DawaPlus 3.0 and DawaPlus 4.0 performed better than the reference net (non-PBO net) in terms of deterrence, induced exophily, blood feeding inhibition and mortality [25]. The dissimilarity in the findings among three countries could be due to the difference in the resistance mechanisms from one mosquito population to another, influenced by the climatic/geographical differences [26]. The authors intend to conclude that DawaPlus 3.0 and DawaPlus 4.0 with their current specification may not be as effective as required to control the resistant vector, *An. culicifacies,* in east-central India. However, further improvement of formulation and treatment technology that ensure adequate bioavailability of pyrethroids and PBO to mosquitoes could enhance the effectiveness of these nets, provided the issue of mechanism of resistance is taken care of.

## Supplementary information

**Additional file 1: Table S1.** Number collected, proportions exiting, blood feeding and blood feeding inhibition of *An. culicifacies* in different experimental arms.

## Data Availability

The datasets generated and/or analysed during the current study are available from the corresponding author on reasonable request.

## Abbreviations

LLIN: long-lasting insecticidal net
PBO: piperonyl butoxide
IRS: indoor residual spraying
WHOPES: World Health Organisation Pesticide Evaluation Scheme
CHC: community health centre
WHO: World Health Organisation
PHD: per hut density
ANOVA: analysis of variance
CI: confidence intervals
IRR: incidence rate ratio
SE: standard error
df: degrees of freedom
