# Supplementary material for: Evaluation of DawaPlus 3.0 and DawaPlus 4.0, deltamethrin–PBO combination nets against pyrethroid-resistant Anopheles culicifacies in experimental huts in India
Source: Malar J. 2020 Jan 23;19:43. doi: 10.1186/s12936-020-3119-x (PMC6979062; doi:10.1186/s12936-020-3119-x)
Supplement: Supplementary file 1 — Additional file 1: Table S1. Number collected, proportions exiting, blood feeding and blood feeding inhibition of An. culicifacies in different experimental arms. [file 12936_2020_3119_MOESM1_ESM.docx]

**Additional file 1. Number collected, proportions exiting, blood feeding and blood feeding inhibition of *An. culicifacies* in different experimental arms**

|  | **Untreated Net** | **DawaPlus 3.0** | **DawaPlus 3.0** | **DawaPlus 4.0** | **DawaPlus 4.0** | **Dawa Plus 2.0** | **Dawa Plus 2.0** |
| --- | --- | --- | --- | --- | --- | --- | --- |
|  |  |  |  |  |  |  |  |
| Number of washes | 0 | Unwashed | 20 | Unwashed | 20 | Unwashed | 20 |
|  |  |  |  |  |  |  |  |
| Total females collected | 264 | 10 | 19 | 12 | 21 | 17 | 31 |
| Geometric Mean females caught/night (95% C.I.) | 5.22^a^ (5.11-5.33) | 0.18^b^ (0.13-0.23)** | 0.35^b^ (0.29-0.41) | 0.21^b^ (0.16-0.26) | 0.37^b^ (0.30-0.43) | 0.29^b^ (0.22-0.35) | 0.59^b^ (0.52-0.65)** |
| % deterrence | - | 96.2 | 92.8 | 95.4 | 92.0 | 93.6 | 88.3 |
|  |  |  |  |  |  |  |  |
| Total females in verandah and exit traps | 63 | 7 | 12 | 7 | 12 | 9 | 18 |
| % exophily (95% C.I.) | 23.9^a^ (18.7-29.0) | 70.0^b^ (41.6-98.4) | 63.2^b^ (41.5-84.8) | 58.3^b^ (30.4-86.2) | 57.1^b^ (36.0-78.3) | 52.9^b^ (29.2-76.7) | 58.1^b^ (40.7-75.4) |
|  |  |  |  |  |  |  |  |
| Total females blood fed | 248 | 0 | 8 | 8 | 14 | 13 | 21 |
| % blood fed (95% C.I.) | 93.9^a^ (91.1-96.8) | 0 (0-0) | 42.1^b^ (19.9-64.3) | 66.7^b^ (40.0-93.3) | 66.7^b^ (46.5-86.8) | 76.5^b^ (56.3-96.6) | 67.7^b^ (51.3-84.2) |
| % blood feeding inhibition | - | 0 | 55.2 | 28.9 | 28.9 | 18.5 | 27.9 |
|  |  |  |  |  |  |  |  |
| Total No. Dead (24H) | 0 | 5 | 6 | 5 | 7 | 3 | 8 |
| % mortality (95% C.I.)^@^ | 0 (0-0) | 50.0^a^ (19.0-81.0) | 31.6^a^ (10.7-52.5) | 41.7^a^ (13.8-69.6) | 33.3^a^ (13.2-53.5) | 17.6^a^ (3.8-43.4*) | 25.8^a^ (10.4-41.2) |
| Corrected for control %^#^ | - | 50.0^a^ (19.0-81.0) | 31.6^a^ (10.7-52.5) | 41.7^a^ (13.8-69.6) | 33.3^a^ (13.2-53.5) | 17.6^a^ (3.8-43.4*) | 25.8^a^ (10.4-41.2) |

Notes: values in the same row sharing a letter superscript do not differ significantly (*P*> 0.05).

*Confidentialintervals (CIs) for percentages are based on normal approximation to binomial distribution, except the Cis marked with asterisk that are based on exact binomial distribution

# Since control mortality was zero, treated mortality is taken as the corrected mortality.

^@^Since mortality was zero with untreated net (negative control), logistic regression analysis was done by keeping the positive control as the reference category.

**Entry differed significantly between these two arms but not with other treated arms.
